# Supplementary material for: Ciliary Rab28 and the BBSome negatively regulate extracellular vesicle shedding
Source: eLife. 2020 Feb 26;9:e50580. doi: 10.7554/eLife.50580 (PMC7043889; doi:10.7554/eLife.50580)
Supplement: Supplementary file 1. [file elife-50580-supp1.docx]

| **Key Resources Table** | | | | |
| --- | --- | --- | --- | --- |
| **Reagent type (species) or resource** | **Designation** | **Source or reference** | **Identifiers** | **Additional information** |
| Genetic reagent (*Caenorhabditis elegans*) | *rab-28(tm2636)* | National Bioresource Project, Tokyo, Japan |  | Wormbase |
| Genetic reagent (*Caenorhabditis elegans*) | *rab-28(gk1040)* | Caenorhabditis Genetics Centre, Minneapolis,  MN, USA | Strain VC2505 | Wormbase |
| Genetic reagent (*Caenorhabditis elegans*) | *bbs-8(nx77)* | Caenorhabditis Genetics Centre, Minneapolis, MN, USA | Strain MX52 | Wormbase |
| Genetic reagent (*Caenorhabditis elegans*) | *bbs-5(gk507)* | Caenorhabditis Genetics Centre, Minneapolis, MN, USA | Strain VC1113 | Wormbase |
| Genetic reagent (*Caenorhabditis elegans*) | *arl-6(ok3472)* | Caenorhabditis Genetics Centre, Minneapolis, MN, USA | Strain RB2509 | Wormbase |
| Genetic reagent (*Caenorhabditis elegans*) | *pdl-1(gk157)* | Caenorhabditis Genetics Centre, Minneapolis, MN, USA | Strain VC282 | Wormbase |
| Genetic reagent (*Caenorhabditis elegans*) | *arl-3(tm1703)* | National Bioresource Project, Tokyo, Japan |  | Wormbase |
| Genetic reagent (*Caenorhabditis elegans*) | N2;oqEx304 [gfp::rab-28 Q95L + unc-122p::gfp] | PMID: 27930654 | Strain OEB803 |  |
| Genetic reagent (*Caenorhabditis elegans*) | him-5(e1490); myIs4 [PKD-2::GFP + Punc-122::GFP] |  | Strain PT621 |  |
| Genetic reagent (*Caenorhabditis elegans*) | him-5(e1490); myIs23[cil-7p::gCIL-7::GFP_3’UTR + ccRFP] |  | Strain PT2679 |  |
| chemical compound, drug | Levamisole hydrochloride | Sigma Aldrich | BP212 |  |
| sequence-based reagent | rab-28(tm2636)_fwd | This paper | PCR primers | CATATGAGGCGTCTGATTGCTGA |
| sequenced-based reagent | rab-28(tm2636)_rev | This paper | PCR primers | GACGTGTGGTTTCAGTTGAAG |
| sequence-based reagent | rab-28(gk1040)_ fwd | This paper | PCR primers | GAATCACGCTTCCACACGAAATGCAG |
| sequenced-based reagent | rab-28(gk1040)_mut_rev | This paper | PCR primers | GGATAAAATTTTTGACGCCGGGATCG |
| sequence-based reagent | rab-28(gk1040)_wt_rev | This paper | PCR primers | CCGGTGAAACTAGTATTGATGGG |
| sequenced-based reagent | pdl-1(gk157)_fwd | This paper | PCR primers | AATTTGTAGGTTATCTAAACGATAC |
| sequence-based reagent | pdl-1(gk157)_mut_rev | This paper | PCR primers | CTTAGAAATCTAGTACTTTTGCAC |
| sequenced-based reagent | pdl-1(gk157)_wt_rev | This paper | PCR primers | GAGCTCTACTCTCTGAAGAAAG |
| sequence-based reagent | arl-6(ok3472)_fwd | This paper | PCR primers | TTGAACTCTTGCCGAAAAGGTC |
| sequenced-based reagent | arl-6(ok3472)_rev | This paper | PCR primers | CTTTGAGCAACTCCATTCGAAG |
| sequence-based reagent | bbs-8(nx77)_fwd | This paper | PCR primers | TTGTGTAGGCGAGACGAAGC |
| sequenced-based reagent | bbs-8(nx77)_mut_rev | This paper | PCR primers | ATTTCAGGGCCCAATCCACC |
| sequence-based reagent | bbs-8(nx77)_wt_rev | This paper | PCR primers | ACAAATACGCAGCCGATCCA |
| sequenced-based reagent | bbs-5(gk507)_fwd | This paper | PCR primers | AGAAGTCATTCTGAGCGTC |
| sequence-based reagent | bbs-5(gk507)_mut_rev | This paper | PCR primers | CTACCGTACACCTGGAC |
| sequenced-based reagent | bbs-5(gk507)_wt_rev | This paper | PCR primers | GATTAGGCGGAGAAGTCTTG |
| sequence-based reagent | arl-3(tm1703)_fwd | This paper | PCR primers | ACATACGGGAGCTAGAGAGT |
| sequenced-based reagent | arl-3(tm1703)_rev | This paper | PCR primers | GTGAGCTGATGAGTGTCTGA |
| sequence-based reagent | rab-28 exon1F | This paper | PCR primers | ATGACAACAATGGGAGAAGAC |
| sequenced-based reagent | rab-28 exon 7R | This paper | PCR primers | TTAAGTAATGGAGCATACAGATG |
| sequence-based reagent | rab-28seqF | This paper | PCR primers | AGATGGAGCAAGTGGAAAGAC |
| software, algorithm | Fiji | PMID:22743772 | RRID:SCR_002285 |  |
